# Supplementary material for: Dynamic transcriptomic profiles of zebrafish gills in response to zinc supplementation
Source: BMC Genomics. 2010 Oct 11;11:553. doi: 10.1186/1471-2164-11-553 (PMC3091702; doi:10.1186/1471-2164-11-553)
Supplement: Additional file 2 — Interactive Direct Interaction Network representing the molecular interactions between zinc, copper, iron, calcium and proteins encoded by transcripts changed by zinc supplementation. Mini web-site containing index.html and hyperlinked pages in subdirectory describing a Direct Interaction Network automatically generated based on curated interactions contained within the proprietary PathwayArchitect database. Ovals represent proteins and the circles symbolize metal ions. Objects are coloured by their abundance in zebrafish at the time-point they were significantly different from the control is a scale from -4 fold (dark green) to +4 fold (dark red). Where significant differences were found at more than one time-point, the colour overlay shows expression at the first instance. Dark blue squares denote 'binding', and light blue squares 'expression'; green squares stand for 'regulation', green diamonds for 'metabolism', and green circles for 'promoter binding'. Arrow heads indicate directionality of the interaction where annotated. All nodes and edges can be further interrogated by selecting the relative area of the image. [file 1471-2164-11-553-S2.zip › PathwayArchitect Zn xs DIN/1593951.html]

# BINDING:

|  |  |
| --- | --- |
| Type | BINDING |
| Effect | None |


---

|  |  |
| --- | --- |
| Score | 0 |


---

|  |  |
| --- | --- |
| Reference Count | 2 |


---

|  |  |
| --- | --- |
| Mechanism | Unknown |


---

|  |  |
| --- | --- |
| Reference:0 || PMID | 15761153 |
| SourceID | 218584 |
| Species | Mouse |
|  | Human |
| Experimental Condition | in-vivo |
| Description | An interaction between human SMAD1 and mouse 1110028N05RIK (homologue of human DKFZP564C103) was demonstrated by co-immunoprecipitation. 3Flag-1110028N05RIK was immunoprecipitated from extracts of HEK-293T cells transfected with hRL-SMAD1 and 3Flag-1110028N05RIK using anti-Flag. hRL-SMAD1 was detected by measuring RL activity on precipitates. Luminescence intensity ratio (LIR) was calculated over the negative control. A LIR of not less than 3 was considered significant. The LIR value of this interaction was 3.249. The false positive rate was 20%. Fig. 2B, Table S1, S2. |
| Detection Method | immunoprecipitation |
| Source | BIND |
  |
|  |  |


---

|  |  |
| --- | --- |
 Reference:1 || PMID | 15761153 |
| Year | 2005 |
| SourceID | 58547 |
| Species | Mouse |
|  | Human |
| Experimental Condition | in-vivo |
| Journal | Science |
| Detection Method | coimmunoprecipitation |
| Source | MINT |
| MINT confidence measure | 1 |
  |


---

|  |  |
| --- | --- |
